# Proteins and Peptides as Important Modifiers of the Polymer Scaffolds for Tissue Engineering Applications—A Review

**DOI:** 10.3390/polym12040844

**Published:** 2020-04-06

**Authors:** Katarzyna Klimek, Grazyna Ginalska

**Affiliations:** Chair and Department of Biochemistry and Biotechnology, Medical University of Lublin, Chodzki 1 Street, 20-093 Lublin, Poland; g.ginalska@umlub.pl

**Keywords:** bioactive construct, biocompatibility, biomolecules, cytotoxicity, ECM, hydrogels, protein carrier, regenerative medicine, stem cells, tissue repair

## Abstract

Polymer scaffolds constitute a very interesting strategy for tissue engineering. Even though they are generally non-toxic, in some cases, they may not provide suitable support for cell adhesion, proliferation, and differentiation, which decelerates tissue regeneration. To improve biological properties, scaffolds are frequently enriched with bioactive molecules, inter alia extracellular matrix proteins, adhesive peptides, growth factors, hormones, and cytokines. Although there are many papers describing synthesis and properties of polymer scaffolds enriched with proteins or peptides, few reviews comprehensively summarize these bioactive molecules. Thus, this review presents the current knowledge about the most important proteins and peptides used for modification of polymer scaffolds for tissue engineering. This paper also describes the influence of addition of proteins and peptides on physicochemical, mechanical, and biological properties of polymer scaffolds. Moreover, this article sums up the major applications of some biodegradable natural and synthetic polymer scaffolds modified with proteins and peptides, which have been developed within the past five years.

## 1. Introduction: The Role of Proteins and Peptides in TE

**Figure 1:**
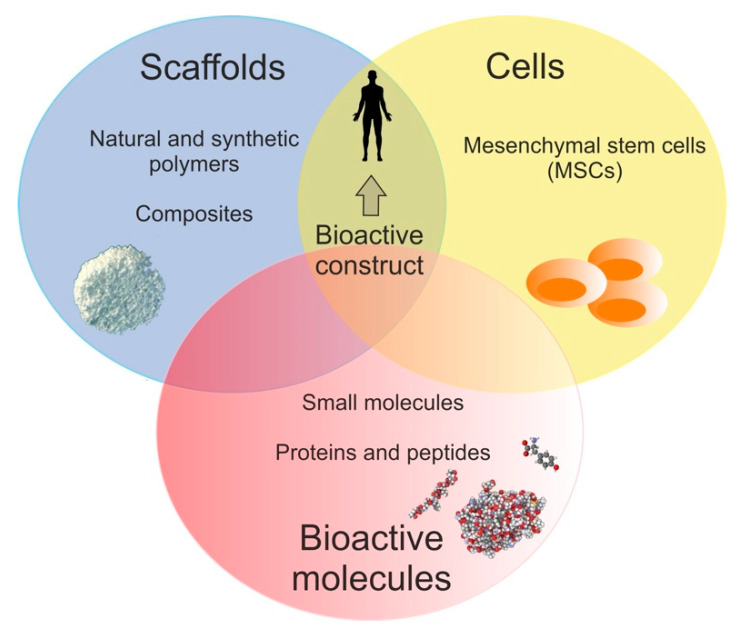
The classical TE paradigm including scaffolds, cells, and bioactive molecules. These three elements may be used alone or in combination. Their association, known as a “bioactive construct”, currently makes up the most successful strategy for tissue repair and regeneration. The choice of appropriate scaffolds (e.g., polymer-based), cells (e.g., primarily stem cells) as well as molecules (especially proteins and peptides) is crucial to carry out an auspicious therapy and tightly depends on future applications.

Tissue engineering (TE) is a multidisciplinary field, which constitutes an alternative and promising approach for grafts, i.e., autografts, allografts, and xenografts [1,2,3]. Thus, TE focuses on providing appropriate solutions, which enable repair or substitute of damaged skin, cartilage, bone, nerves, bladder, blood vessels, or heart valves [4,5]. The classical TE involves scaffolds, cells, and bioactive molecules alone or the association of these three elements. The combination of scaffold, cells, and bioactive molecules—a bioactive construct—is currently considered as the best option for tissue repair and regeneration [5,6,7,8]. The main assumptions of the classical TE are presented in Figure 1.

Suitable scaffolds play a pivotal role in the classical TE strategy. Scaffolds are three-dimensional (3D) matrices, which mimic native extracellular matrix (ECM) to support cell growth. Therefore, they should be biocompatible, biodegradable at the desired rate, porous, and mechanically stable [6,9,10,11]. Considering these requirements, the scaffolds based on natural and synthetic polymers alone, as well as composites, exhibit the highest biomedical potential [12,13,14]. Among known biodegradable natural polymers, collagen, gelatin, chitosan, elastin, hyaluronic acid (HA), and silk fibroin are commonly used for TE purposes [15,16]. In turn, poly-ε-caprolactone (PCL), polylactic acid (PLA), poly-glycolic acid (PGA), and poly(lactic-co-glycolic acid) (PLGA) have attracted great attention in the group of biodegradable synthetic polymers [17,18]. Nevertheless, both natural and synthetic polymers have advantages and drawbacks. For instance, natural polymers are usually biocompatible, but exhibit poor mechanical properties, whereas synthetic ones are generally characterized by good mechanical strength and relatively low biocompatibility [1,4,10,11,19]. Biocompatibility is one of the most important features of scaffolds, because it allows for proper interactions between cells and scaffold. It means that biocompatible biomaterials are non-toxic and they enable excellent cell adhesion, spreading, proliferation, and differentiation [10,13,18,19]. In some cases, the polymer scaffolds do not exhibit toxic effect, but the properties of their surface (e.g., topography, wettability, charge) do not promote cell adhesion, proliferation, and differentiation. Such biomaterials should be modified (e.g., with bioactive molecules) in order to increase their biocompatibility [6,11,20,21].

Apart from scaffolds, cells also take an important place in classical TE paradigm. To select adequate kind of cells, practical, ethical, and biological aspects need to be included. Recently, it is considered that adult mesenchymal stem cells (MSCs) constitute a “gold standard” for regenerative medicine [19,22,23]. Given available sources of MSCs, they are mainly isolated from bone marrow (BMSCs) as well as adipose tissue (ASCs) [24,25,26]. The MSCs possess capacity to self-renew and differentiate into various cells, such as adipocytes, chondrocytes, fibroblasts, myocytes, and osteoblasts [25,26]. In the ex vivo strategy, including inter alia fabrication of bioactive construct, the isolated cells need suitable support for growth (scaffold) and biological signals (mainly growth factors), which promote their growth, proliferation, and differentiation into desired tissue type [25,27,28,29].

The bioactive molecules—constituting the third pillar in classical TE—have a great influence on both scaffold properties and cell behavior. From the TE point of view, bioactive molecules are biological factors or signals that improve properties of scaffolds, support cellular activities, and as a consequence lead to better and intensive regeneration of specific tissue [6,18,19]. Thus, scaffold enrichment with bioactive molecules primarily promotes its biocompatibility, by leading to increased cell adhesion, proliferation, and differentiation. It is worth noting that proteins, especially growth factors (GFs), are currently ones of the most applied bioactive molecules in classical TE [9,18,19,30,31,32]. Besides growth factors, ECM proteins, adhesive peptides, hormones, cytokines, or some enzymes have favorable influence on scaffold biocompatibility [6,9,18,19,33].

Given the high significance of bioactive molecules in classical TE paradigm, this review characterizes the most important proteins and peptides, which are used as modifiers of polymer scaffolds. This article also elucidates the influence of proteins/peptide addition on physicochemical, mechanical, and biological properties of these biomaterials. Moreover, this review summarizes major biomedical applications of biodegradable natural and synthetic polymer scaffolds modified with proteins and peptides.

## 2. Characterization of Proteins and Peptides Used as Modifiers of Polymer Scaffolds

As mentioned above, scaffolds act as support for cell anchorage and thus their structure as well as composition should be the same as natural ECM. Native ECM constitutes not only a physical reinforcement for cell growth, but also regulates many cellular processes, inter alia cell survival, migration, and differentiation [34,35,36,37]. The ECM is mainly composed of fibrous proteins (various types of collagen and elastin), adhesive glycoproteins (i.e., fibronectin, laminin, and vitronectin), proteoglycans (PGs), and glycosaminoglycans (GAGs). Apart from listed components, growth factors also occur within ECM and play pivotal role as cellular modulators [34,35,36]. Thus, improvement of polymer scaffolds with proteins (especially ECM proteins and/or growth factors) and peptides enables fabrication of biocompatible biomaterials that mimic natural ECM [38,39,40,41].

### 2.1. Collagen

Collagen is the most abundant protein in ECM of different tissues. Thus far, nearly 30 types of collagen have been identified and well-characterized [34,36,42]. Nevertheless, only some types of collagen occur in ECM (i.e., Types I, II, III, V, and XI) and have ability to form stretch-resistant fibers [35,37,42,43]. For example, collagen Type I constitutes the major component of most connective tissues, especially tendons [34,44], skin [44,45], and bone [44,46], while collagen Type II predominantly exists in cartilage [44,47]. In turn, ECM of blood vessels mainly contains collagen Type III [34]. Each types of collagen possess identical structure, which is composed of three α-chains. The α-chains comprise at least one repeating sequence –[Gly-X-Y]_n_, where X and Y are usually proline and hydroxyproline, respectively. These α-chains spontaneously form left-handed helices, which in turn create right-handed triple helix. In animals and humans, triple helices generate more complex structures—fibrils and then fibers [43,48,49]. The presence of collagen fibers in ECM primarily provides tensile strength but also regulates cell adhesion, growth, proliferation, and differentiation. Thus, many cells, such as fibroblasts, osteoblasts, and chondrocytes, have ability to synthesize and secrete this protein [34,36,37,47,50].

Collagen for biomedical applications is most often isolated from bovine skin and tendons, porcine bladders, rat tail tendons, fish, and sea plants [44,48,51]. It is biocompatible and biodegradable as well as exhibits relatively low inflammatory host response. It is worth noting that the fibrillar types of collagen (especially Type I) are usually applied for production of collagen-based biomaterials [34,43,44,52]. Nevertheless, extraction of pure form of collagen Type I is associated with very high costs. Despite this fact, up to date, many biomaterials composed of collagen alone have been fabricated [42,43,51,52,53,54,55,56]. Such biomaterials possess high biocompatibility, but their uses are limited because of poor mechanical properties and ability to degrade in a relatively short time [57,58,59]. For these reasons, collagen is widely applied as a modifier of biodegradable natural and synthetic polymer scaffolds. Combination of collagen with other polymers allows obtaining biocompatible and mechanically stable biomaterials for TE applications [48,50,52,56,58].

### 2.2. Gelatin

Gelatin is a natural mixture of polypeptides that is obtained from collagen Type I. Collagen using for gelatin extraction may be isolated from bones, tendons, and skin of animals, but it is especially obtained from bovine and porcine [60,61]. To date, two main methods for gelatin extraction have been developed. One of them includes heat treatment of collagen. This process breaks hydrogen bonds, destabilizes collagen triple helix structure, and as a consequence converts it to a coiled form of gelatin. Gelatin may also be received during partial hydrolysis of collagen. Depending on hydrolysis conditions, two types of gelatin are produced. Generally, gelatin Type A is obtained in acidic solutions, while gelatin Type B is received in alkaline ones. For this reason, both types of gelatin possess different features, such as amino acids composition, charge, and isoelectric point. It is worth noting that either types of gelatin are commercially available at relatively low cost [50,61,62,63].

Gelatin exhibits many biological similarities to collagen. Primarily, both polymers are biocompatible, biodegradable, and abundant in RGD sequences (Arg-Gly-Asp) that promote cell adhesion. Moreover, they have the same characteristic amino acids sequence –[Gly-X-Y]_n_, where X and Y mostly constitute proline and hydroxyproline. However, in contrast to collagen, gelatin does not induce antigenicity. This phenomenon is tightly associated with no or low presence of aromatic amino acids (i.e., tyrosine, tryptophan, and phenylalanine) in gelatin molecule. Thus, gelatin is found to form significantly lower amount of immunogenic aromatic radicals compared to collagen [14,50,64,65,66].

Thanks to superior biological properties, gelatin has been extensively applied in TE [61,66,67,68,69]. Nevertheless, it also possesses disadvantages, such as poor mechanical strength, high viscosity, fast enzymatic degradation, and reduced solubility. Thus, similarly to collagen, gelatin application alone is limited. For this reason, gelatin is combined with other polymers in order to enhance its mechanical properties as well as to promote biocompatibility of these polymers [50,61,62,66,67].

### 2.3. Elastin

Elastin is a fibrous ECM protein that is composed of single tropoelastin subunits. The main role of elastin is to provide elasticity and resilient to many connective tissues [40,70,71]. Its elasticity is coded by a repeating amino acids sequence—VPGVP, where V, P, and G are valine, proline, and glycine, respectively. Elastin and microfibrils create elastic fibers, which are remarkably important elements in vascular and connective tissues as well as organs (i.e., skin, lungs, heart, and bladder). Besides providing elasticity, elastin supports cell adhesion and growth due to presence of RGD sequences in its molecule [34,35,43,71].

Although elastin is a key structural ECM protein, it is not used as frequently as other ECM proteins (e.g., collagen, fibronectin, and laminins) in engineering of biomaterials, primarily because of the arduous process to purify elastin. During this process, some contaminations may occur. Consequently, their presence may lead to immune response by body [15,40,43]. For this reason, artificial forms of elastin, i.e., synthetic tropoelastin, α-elastin, elastin-like polypeptides (ELPs), and elastin-like recombinamers (ELRs), have been developed. Thanks to their high elasticity, water-solubility, biocompatibility, and biodegradability, they currently constitute good alternatives for elastin [14,15,38,40,70].

### 2.4. Adhesive Glycoproteins

The adhesive glycoproteins, i.e., fibronectin, laminins, and vitronectin, play a key role in supporting cell migration and adhesion to ECM. Among these proteins, fibronectin and laminins are mainly used for modification of both natural and synthetic biomaterials in order to improve their biocompatibility [3,6,7,9,12,15,18,19,72,73,74].

#### 2.4.1. Fibronectin

Besides collagen, fibronectin is the most important protein that occurs in ECM. Moreover, another form of this protein is a component of blood plasma [34,35,36]. Both the plasma and cellular forms usually consist of two almost the same subunits, which are composed of three distinct types of repeating modules (Types I–III). The main role of this glycoprotein is to enhance cell migration, adhesion, spreading, and proliferation, but it is also necessary for the development of many tissues and organs during embryogenesis [37,38,75]. It also plays important roles in cellular morphology and wound healing. The significant role of fibronectin in enhancement of cell adhesion results from its capacity to bind to α_5_β_1_ integrin of cell membrane via RGD sequence. Furthermore, it has the ability to tie with PGs, other adhesion proteins, and GFs.

#### 2.4.2. Laminins

Laminins constitute a big family of cross-shaped glycoproteins with trimetic structure composing of α, β, and γ chains. Interactions between these proteins create space for adhesion between different tissues [34,36,76]. Laminins, together with collagen Type IV, nidogens, agrin, and perlecan, occur in basement membrane (MB) of ECM. Nevertheless, laminins mainly dictate the structure and assembly of MB [77]. Moreover, these glycoproteins play a crucial role in cell migration, adhesion, differentiation, and wound healing [78,79,80,81]. They also participate in early embryonic development and organogenesis. Laminins and laminin-derived peptides are successfully used for enrichment of biomaterials [38,75,82,83].

### 2.5. Growth Factors

Growth factors (GFs) are signaling molecules that transmit signals to modulate cellular activities [19,25,72,84]. The GFs are produced by many cells, which are involved in regenerative processes. These proteins can also be sequestered by ECM for presentation of cell surface receptors. Growth factors can act via autocrine, paracrine, and endocrine mechanisms [32,84,85,86]. When GFs reach a suitable concentration, the reparation process begins. Thus, these soluble proteins stimulate cell migration, growth, proliferation, differentiation, and gene expression. They play important roles in wound healing, tissue regeneration, and immune regulation. Nevertheless, these proteins possess short effective half-life, low stability, and they are inactivated by enzymes under physiological conditions [87,88,89,90]. Combination of the GFs with suitable biomaterial allows for preservation of GFs activity and enables their sustained release. Among the many known growth factors, bone morphogenetic proteins (BMPs), insulin-like growth factor-1 (IGF-1), transforming growth factor-β (TGF-β), basic fibroblast growth factor-2 (FGF-2), platelet-derived growth factor (PDGF), and vascular endothelial growth factor (VEGF) are most commonly used for TE applications [19,25,32,36,72,73,84,85,91,92,93]. This review focuses on three of them, i.e., BMPs, FGF-2, and VEGF.

#### 2.5.1. Bone Morphogenetic Proteins (BMPs)

Bone morphogenetic proteins are a group of regulatory glycoproteins that belong to the large super-family of transforming growth factors-β (TGF-β) [87,88,94]. To date, approximately 20 BMP members have been identified. They are divided into four families: (1) BMP-2 and BMP-4; (2) BMP-3 and BMP 3B; (3) BMP-5, BMP-6, BMP-7, and BMP-8; and (4) GDF-5, GDF-6, and GDF-7. BMPs play crucial roles in the growth of mesenchymal cells and promote their differentiation into chondrocytes and osteoblasts [90,92]. They also promote maintenance of a chondrocyte phenotype and enhance upregulation of cartilage matrix synthesis. BMPs have important roles in tooth development, differentiation of odontoblasts, and development of cement and alveolar bone. Moreover, they regulate chemotaxis, alkaline phosphate activity, and osteocalcin synthesis/mineralization [31,90,92,95]. Considering their osteogenic potential, BMPs are successfully used in many therapeutic interventions including bone defects or osteoporosis. For instance, recombinant human BMP-2 (rhBMP-2) combined with collagen sponge (INFUSE Bone Graft) is used for the treatment of spinal fusion and tibial fractures, whereas recombinant human BMP-7 (rhBMP-7), marketed as OP-1 Putty, is used for lumbar spine fusion and the treatment of bone fractures [95,96,97,98].

#### 2.5.2. Fibroblast Growth Factor-2 (FGF-2)

Fibroblast growth factors family includes approximately 22 polypeptides that can improve survival, migration, proliferation, differentiation, and metabolic activity of different cells [99]. They are secreted by keratinocytes, fibroblasts, endothelial cells, smooth muscle cells, chondrocytes, and mast cells. Moreover, they promote angiogenesis and wound healing. The FGFs are arranged into seven subfamilies [100]. Basic fibroblast growth factor-2 (FGF-2) is produced by fibroblasts and endothelial cells [86]. It primarily simulates cell proliferation and differentiation [73,91]. Moreover, FGF-2 affects angiogenesis, adipogenesis, wound healing, and tissue repair [95]. FGF-2 combined with scaffolds is used for regeneration of damaged skin, blood vessel, muscle, cartilage, bone, and nerve [92].

#### 2.5.3. Vascular Endothelial Growth Factor (VEGF)

Vascular endothelial growth factor is secreted by fibroblasts, mast cells, keratinocytes, macrophages, and platelets [86,90,92,95]. It induces migration and proliferation of endothelial cells, which improves the mitogenic response to angiogenic factors. It also improves epidermal repair and formation of granulation tissue. VEGF is known to promote bone repair, while its absence leads to inhibition of bone regeneration [16,88,91,99,101].

### 2.6. Decellularized Extracellular Matrix (dECM)

Decellularized extracellular matrix (dECM) is a promising biomaterial for tissue engineering applications, because it retains properties of natural tissues or organs, such as composition, architecture, and integrity, as well as biochemical and biological activities [9,102]. Similar to native ECM, dECM consists of heterogeneous mixture of proteins (i.e., collagen, laminins, fibronectin, and GFs), PGs, and GAGs, and thus it constitutes a proper template for cell adhesion, proliferation, and differentiation [103,104]. Nevertheless, the dECM is deprived of cellular components, which decreases an inflammatory response in the host organism [9,105]. To derive dECM from tissues, perfusion decellularization is most often used. It mainly involves chemical and enzymatic lysis of cells and then vascular perfusion in order to remove cell debris. In contrast to physical methods, this procedure seems to be more favorable, because it minimizes the ECM damage [104,105,106]. To date, dECM from heart, lungs, kidneys, urethra, trachea, bones, cartilage, and bladders has been obtained [9,103,105,106,107,108]. However, limited number of autologous tissues/organs and risk of infection associated with allogenous as well as xenogenous ones still represent big problems for scientists [109]. Besides tissues and organs, in vitro cell cultures make up an alternative source of the dECM [9,109,110,111]. To obtain cell-derived dECM, chemical or enzymatic treatment of cultured MSCs or other adult cells (e.g., fibroblasts) are used [102,109,110,112]. Cell-derived dECM possesses many advantages in comparison with the dECM obtained from tissues/organs. Primarily, it has safe origin (cultured cells might be checked for pathogens) and it can be prepared from many kinds of autologous cells (cells are isolated from patient organism and cultured in laboratory) [109,111,113]. Nevertheless, the procedures necessary for production of cell-derived dECM are usually expensive and time-consuming [9]. Despite all these limitations, dECM derived from tissues/organs as well as cell cultures is successfully used either alone or in combination with polymers [105,106,108,109,112,113].

### 2.7. Peptides

Apart from proteins, peptides are a very important modifiers of polymer scaffolds. Thanks to beneficial properties of peptides, there is an increasing trend to modify biomaterials with these small molecules [38,114,115]. It was found that peptide-modified biomaterials exhibit structural and biological properties close to those of protein-modified scaffolds. Nevertheless, peptides possess more advantages than proteins. For instance, production of peptides is simpler and more cost-effective compared to the fabrication process of full-length proteins. Likewise, modification of peptides is significantly easier compared to alteration of high-molecular weight proteins. Moreover, peptides are more resistant to environment conditions (e.g., pH and temperature) and they are relatively safe due to a low immunogenicity. To date, many peptides that mimic functions of ECM proteins and some growth factors have been identified. Combination of these peptides with polymer scaffolds results in an enrichment of biomaterials with characteristic sequences that promote cell adhesion or induce cell signaling pathways [38,114,116,117].

#### 2.7.1. Peptides Derived from Collagen, Fibronectin, and Laminins

Among known peptides able to enhance cellular activities, RGD (Arg-Gly-Asp) are considered as the most important modifier of biomaterials. As mentioned above, this pro-adhesive motif occurs mainly in collagen, gelatin, elastin, fibronectin, and laminins. RGD makes up an anchoring place for both α and β integrin receptors, which enhances adhesion and proliferation of many kind of cells [38,115,117,118].

It is worth noting that there are several, different from RGD, pro-adhesive sequences coming from collagen. Among these, the most noteworthy are DGEA (Asp-Gly-Glu-Ala), GFOGER (Gly-Phe-HPro-Gly-Glu-Arg), and GFPGER (Gly-Phe-Pro-Gly-Glu-Arg). Similar to collagen, the mentioned peptides bind to α2β1 integrin and can promote cell adhesion, proliferation, and differentiation [38]. On the other hand, peptide PepGen P-15 (P-15) also constitutes a very interesting pro-adhesive motif derived from collagen. This peptide comprises –GTPGPQGIAGQRGVV– (-Gly-Thr-Pro-Gly-Pro-Gln-Gly-Ile-Ala-Gly-Gln-Arg-Gly-Val-Val-) amino acid sequence, which is the same as the one existing in the cell-binding region of collagen Type I. P-15 is known to stimulate osteoblast adhesion and proliferation. Moreover, P-15 upregulates osteogenic gene expression of runt-related transcription factor-2 (RUNX2), osterix (OSTRX), and bone sialoprotein (BSP) [117,118,119].

In the case of fibronectin derived sequences, the PHSRN (Pro-His-Ser-Arg-Asn), REDV (Arg-Glu-Asp-Val), LDV (Leu-Asp-Val), and KQAGDV (Lys-Gln-Ala-Gly-Asp-Val) are usually used in TE. Similar to collagen-derived sequences, they also possess the ability to bind to integrin receptors resulting in enhanced adhesion and proliferation of many cells, such as fibroblasts, MSCs, and endothelial cells [38,120,121,122,123].

Another important integrin binding ligands come from laminins. Thus, IKVAV (Ile-Lys-Val-Ala-Val) and YIGSR (Tyr-Ile-Gly-Ser-Arg) are considered as the most promising pro-adhesive motifs [38,75,82,83]. Apart from these pro-adhesive sequences, the pro-angiogenic peptide C16 is also derived from laminins. It comprises KAFDITYVRLKF (Lys-Ala-Phe-Asp-Ile-Thr-Tyr-Val-Arg-Leu-Lys-Phe) amino acid sequence and enhances endothelial cell migration, adhesion, and proliferation. Moreover, it can support angiogenesis in vivo [124].

#### 2.7.2. BMPs-Derived Peptides

It is well known that BMPs play a crucial role in the formation of bone [90,92]. For this reason, several BMPs-derived peptides have been developed. These peptides mainly come from BMP-2 and BMP-7 [117]. In the case of BMP-2-derived peptides, it is worth underlining that peptides P17 and P24 are the most promising for bone TE applications. The peptide P17—IVAPPGYHAFYCHGECP (Ile-Val-Ala-Pro-Pro-Gly-Tyr-His-Ala-Phe-Tyr-Cys-His-Gly-Glu-Cys-Pro)—enhances viability of BMSCs, simulates osteogenic gene expression, and accelerates new bone formation in vivo [125]. Likewise, peptide P24—KIPKASSVPTELSAISTLYLSGGC (Lys-Ile-Pro-Lys-Ala-Ser-Ser-Val-Pro-Thr-Glu-Leu-Ser-Ala-Ile-Ser-Thr-Leu-Tyr-Leu-Ser-Gly-Gly-Cys)—supports adhesion of BMSCs, osteogenic gene expression as well as bone regeneration [126]. In turn, peptide BFP-1 (derived from BMP-7) comprises GQGFSYPYKAVFSTQ (Gly-Gln-Gly-Phe-Ser-Tyr-Pro-Tyr-Lys-Ala-Val-Phe-Ser-Thr-Gln) amino acid sequence, which supports BMSCs differentiation in vitro and bone formation in vivo. Importantly, this peptide exhibits higher osteogenic potential than BMP-7 [127]. Moreover, another BMP-7 mimetic peptide containing KQLNAISVLYFDD (Lys-Gln-Leu-Asn-Ala-Ile-Ser-Val-eu-Gln-Phe-Asp-Asp) amino acid sequence improves bone regeneration in vivo [128].

#### 2.7.3. Peptide QK

VEGF is known as a crucial regulator of angiogenesis [38,116]. QK is the most well-characterized, pro-angiogenic peptide that mimic activity of VEGF. This molecule contains KLTWQELYQLKYKGI (Lys-Leu-Thr-Trp-Gln-Glu-Leu-Tyr-Gln-Leu-Lys-Tyr-Lys-Gly-Ile) amino acid sequence, which imitates the α-helix region of VEGF. For this reason, QK has high affinity to bind to VEGF receptors on the surface of endothelial cells [38,116]. Many studies have revealed that peptide QK promotes endothelial cell migration, viability, and proliferation in vitro [129,130,131,132,133] as well as supports angiogenesis in vivo [130,133,134].

#### 2.7.4. Peptide RADA-16-I

The peptide RADA-16-I is comprised of -RADARADARADARADA- (-Arg-Ala-Asp-Ala-Arg-Ala-Asp-Ala-Arg-Ala-Asp-Ala-Arg-Ala-Asp-Ala-) amino acid sequence. This peptide belongs to class of self-assembly peptides (SAPs), which amino acids have tendency to spontaneous adopt a β-sheet structure in the presence of monovalent cation solutions or under physiological conditions. Such process results in formation of self-assembled matrices with interwoven nanofibers [117,118]. It was demonstrated that RADA-16-I enhances adhesion and proliferation of keratinocytes, fibroblasts [135], and osteoblasts [136,137]. Moreover, the RADA-16-I is known to simulate osteogenic differentiation of BMSCs via upregulation of ALP (alkaline phosphatase), RUNX2, and OC (osteocalcin) gene expression.

## 3. Influence of Proteins/Peptides Addition on Properties of Polymer Scaffolds

**Figure 2:**
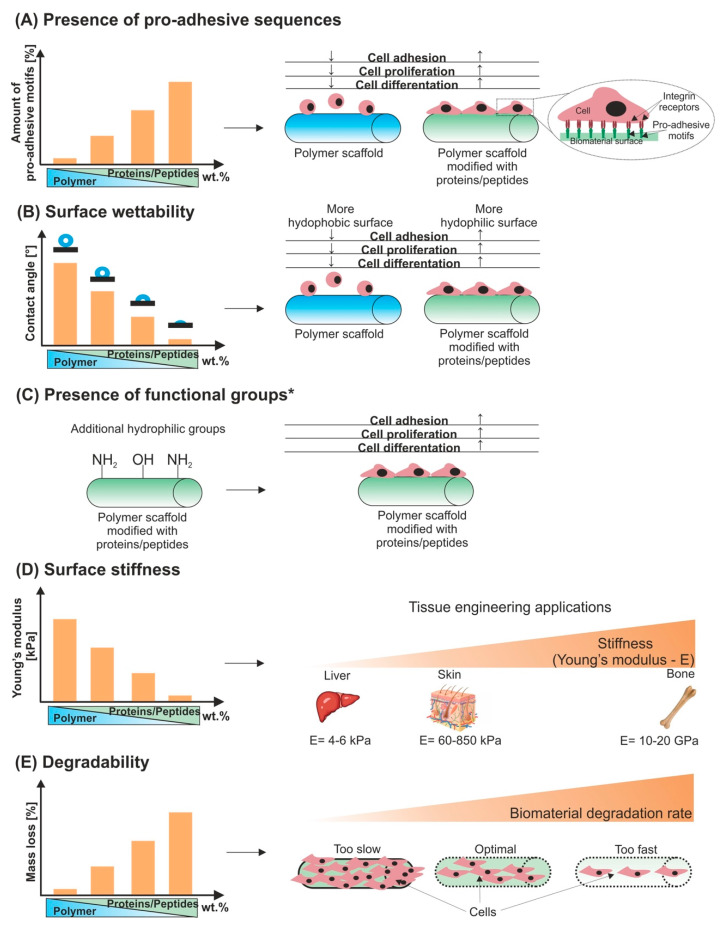
Influence of proteins/peptides addition on properties of polymer scaffolds. Inclusion of these molecules to polymer biomaterials allows for: (**A**) introduction of pro-adhesive sequences, which promote cell adhesion, proliferation, and differentiation; (**B**) enhancement of hydrophilicity of biomaterial surface, which improves cell–biomaterial interactions; (**C**) introduction of additional functional groups (e.g., –NH_2_ or –OH groups), which support cellular activity (* presence of additional free functional groups depends upon interactions between functional groups of proteins/peptides and chemical moieties of polymers); (**D**) adjustment of surface stiffness to obtain an appropriate scaffold for TE applications; and (**E**) adjustment of biomaterial degradation rate that should be correlated with the rate of ECM production by the cells.

To enhance biocompatibility of polymer scaffolds, they are often modified with proteins and/or peptides. Inclusion of these molecules to polymer scaffolds primarily results in enrichment of biomaterial with pro-adhesive sequences. Furthermore, proteins/peptides addition to polymer scaffolds affects their physicochemical features (e.g., surface wettability and charge), mechanical properties, and degradability. These characteristics, in turn, tightly influence the biological activity of the biomaterials [21,138,139]. The influence of proteins/peptides addition on biological, physicochemical, and mechanical properties of polymer scaffolds is presented in Figure 2A–E.

### 3.1. Presence of Pro-Adhesive Sequences

Most proteins (e.g., collagen, gelatin, fibronectin, or laminins) and some peptides contain pro-adhesive sequences, such as RGD, PHSRN, YIGSR, or IKVAV (see Section 2). Combination of these proteins/peptides with polymer scaffolds results in enrichment of biomaterial surface with beneficial binding sites (Figure 2A). It is especially important, because they are recognized by many kinds of cells (mainly via integrin receptors on the cell membrane). Consequently, such recognition promotes cell attachment, proliferation, differentiation [38,117,139].

For instance, Mobasseri et al. [140] showed that nanofibrous PCL scaffold enriched with RGD promoted human BMSC adhesion and proliferation. The authors also proved that this biomaterial allowed maintaining the proper function of BMSCs during long-term culture. Kim et al. [141] demonstrated that PHSRN–gelatin mixture supported adhesion, spreading, and proliferation of human MSCs. Ouyang et al. [142] indicated that polyethylene glycol (PEG) hydrogel modified with RGD promoted adhesion and proliferation of rat BMSCs. Hong and Song [143] demonstrated that 3D PEG hydrogel combined with RGD enhanced adhesion, survival, and osteogenic differentiation of mouse MSCs. Patrulea et al. [144] fabricated chitosan scaffold enriched with RGD and showed that it enhanced adhesion and proliferation of human dermal fibroblasts. Desseaux and Klok [145] proved that polymer-based brush modified with RGD or PHSRN promoted adhesion and proliferation of mouse fibroblasts. Cringoli et al. [146] demonstrated that LDV-based hydrogel supported fibroblast adhesion and survival. Garcia et al. [147] showed that functionalization of HA hydrogels with RGD or IKVAV resulted in enhancement of migration and proliferation of mouse myoblasts. Moreover, Zhou et al. [148] showed that PCL-based membrane combined with REDV promoted adhesion and proliferation of endothelial cells in vitro. Mann and West [149] fabricated PEG scaffold modified with KQAGDV and demonstrated that such biomaterial enhanced migration, adhesion, and proliferation of smooth muscle cells derived from rat thoracic aorta. In another study, Motta et al. [150] presented that RGD, IKVAV, and YIGSR peptides could promote migration, adhesion, and growth of Schwann cells. The authors suggested that these sequences may be useful for modification of scaffolds for nerve regeneration. Li et al. [151] produced biosynthetic extracellular matrix enriched with YIGSR and demonstrated that it promoted regeneration of corneal epithelium and nerves in vivo. Zhang et al. [152] fabricated RGD-decorated macroporous PEG hydrogel and showed that this biomaterial promoted adhesion and viability of primary chondrocytes in vitro. Some studies also indicated that RGD sequence enhanced osteoblast adhesion and proliferation as well increased osteogenic gene expression (i.e., ALP, OC, and OP (osteopontin)), which supported differentiation and mineralization of these cells [38,117,153].

### 3.2. Surface Wettability

Surface wettability is considered as the most important feature that regulates biomaterial behavior in the contact with fluids. Considering the contact angle (θ) measurements, the scaffold surface may be hydrophilic (0° ≤ θ < 90°) or hydrophobic (90° ≤ θ ≤ 180°) [154,155,156]. During in vitro and in vivo conditions, biomaterials have contact with liquids that contain proteins (e.g., bovine serum albumin in complete culture medium; and albumin, fibronectin, laminin, or vitronectin in physiological fluids). It was proved that cells do not interact directly with biomaterial surface but they adhere to the layer of adsorbed proteins. Attached proteins that a have hydrophilic nature increase surface wettability of the biomaterial. Thus, hydrophilic surfaces are considered as more biocompatible than hydrophobic ones [12,21,139,156,157,158,159].

Many studies have demonstrated that the addition of proteins/peptides to polymer scaffolds increases surface hydrophilicity and as a consequence enhances cell adhesion, proliferation, and differentiation (Figure 2B). Sadeghi et al. [160] demonstrated that addition of collagen to PLGA scaffold led to drastically decrease in value of contact angle (from 132° to approximately 0°). The authors also showed that surface of PLGA/collagen was more favorable for cell adhesion than surface of PLGA biomaterial (studies on HDF and HaCaT cell lines). Likewise, Sousa et al. [161] showed that surface of PCL/collagen biomaterial was more hydrophilic (θ = 49.5°) compared to surface of PCL alone (θ = 76.5°). They also proved that the surface of PCL/collagen scaffold enhanced fibroblast proliferation (studies on NIH/3T3 cell line). Shin et al. [162] fabricated PLGA/collagen/graphene oxide biomaterial and demonstrated that surface of tri-component scaffold possessed higher wettability compared to surface of PLGA/graphene oxide composite (contact angle equal to 85° and 126°, respectively). Moreover, the authors indicated that surface of PLGA/collagen/graphene oxide scaffold was more beneficial for skeletal myoblast adhesion, proliferation, and differentiation in comparison with surface of PLGA/graphene oxide biomaterial. In turn, Prado-Prone et al. [163] proved that contact angle decreased, when the content of gelatin in PCL scaffold increased. Thus, the contact angles of PCL, PCL/30 wt.% gelatin, and PCL/45 wt.% gelatin biomaterials were approximately 127°, 65°, and 25°, respectively. The cell culture experiments showed that PCL/45 wt.% gelatin biomaterial exhibited the highest biocompatibility in vitro compared to other scaffolds. Won et al. [164] demonstrated that immobilization of fibronectin onto the surface of PCL scaffold notably decreased value of contact angle. The authors also proved that the number of rat MSCs attached to the surface of PCL/fibronectin scaffold (θ = 31°) was significantly higher compared with number of cells adhered to surface of PCL biomaterial (θ = 80°). Bahrami et al. [165] fabricated nanofibrous PCL scaffold modified with laminin and showed that composite biomaterial possessed better wettability (θ = 64°) compared to PCL alone (θ = 112°). Moreover, the authors proved that PCL/laminin biomaterial accelerated wound healing in vivo (studies on male Wistar rats). Mobasseri et al. [140] showed that surface of nanofibrous PCL/RGD biomaterial possessed higher wettability in comparison with surface of nanofibrous PCL scaffold (θ was approximately 53° and 144°, respectively). Thus, compared to PCL scaffold, PCL/RGD biomaterial promoted human MSC adhesion and proliferation. Wang et al. [166] fabricated op-HAp/PLGA and P15/OPG/pDA/op-HAp/PLGA scaffolds. They showed that the addition of two peptides (P15 and OPG) to PLGA-based biomaterial led to a significant increase in surface wettability (θ decreased from 80° to approximately 18°), which improved adhesion, proliferation, and osteogenic differentiation of mouse preosteoblasts (MC3T3-E1 cell line).

On the other hand, it was found that hydrophobic surfaces can bind a higher amount of proteins than hydrophilic ones. However, in the case of adhesive glycoproteins (i.e., fibronectin, laminin, and vitronectin), there is an exception to this rule, because they exhibit greater affinity to hydrophilic surfaces. Moreover, unlike hydrophobic surfaces, the hydrophilic ones maintain proper conformation of adsorbed proteins, which promotes cell adhesion and proliferation [21,138,158,167,168]. Unfortunately, the above-mentioned papers [140,160,161,162,163,164,165,166] do not present experiments that evaluate biomaterial ability to adsorb proteins from surrounding liquids. In turn, Lȕ et al. [169] fabricated chitosan/collagen film and demonstrated that its surface was more hydrophilic (θ = 64°) compared to surface of chitosan biomaterial (θ = 86°). The Bradford protein assay revealed that both biomaterials possessed similar capacity to adsorb proteins from complete culture medium (high glucose DMEM supplemented with 10% fetal bovine serum (FBS)). Meanwhile, cell culture experiments showed that the number of cells grown on chitosan/collagen biomaterial was significantly higher in comparison with number of cells cultured on chitosan film (studies on PC-12 cell line). It is worth noting that polymer scaffolds modified with proteins/peptides not only can adsorb proteins from surrounding liquids but may also release incorporated proteins/peptides outside. Thus, the quantitative evaluation (mainly using colorimetric tests) of amount of adsorbed proteins by such biomaterials is complicated and may lead to obtain unreliable results. In this case, the qualitative analysis (e.g., using fluorescence dying) seems to be more appropriate for assessment of biomaterial ability to adsorb proteins. Yang et al. [170] fabricated PLGA and PLGA/HAp/collagen fibrous scaffolds. They showed that surface of PLGA/HAp/collagen biomaterial was characterized by lower value of contact angle (θ = 84°) compared to surface of PLGA alone (θ = 100°). Importantly, the fluorescence microscope observation showed that PLGA/HAp/collagen scaffold exhibited a significantly higher ability to adsorb rhodamin labeled fibronectin (Rhodamin-FN) compared to PLGA biomaterial. Moreover, the cell culture experiments proved that hydrophilic surface of PLGA/HAp/collagen biomaterial was better for adhesion, proliferation, and osteogenic differentiation of rat BMSCs in comparison with the hydrophobic surface of PLGA biomaterial.

Regarding the above-mentioned results, it is clear that the addition of proteins/peptides to polymer scaffolds increases surface wettability and promotes biocompatibility. Nevertheless, the ability to adsorb proteins by such biomaterials needs to be better studied.

### 3.3. Presence of Functional Groups

Presence of functional groups is another important feature, which determines biocompatibility of scaffolds. Many researchers have demonstrated that functional groups have high influence on surface wettability, ability to adsorb proteins, and consequently cell behavior (studies using model surfaces, i.e., self-assembled monolayers (SAMs)) [171,172,173,174,175]. For instance, Keselowsky et al. [171] fabricated SAMs functionalized with –CH_3_, –NH_2_, –COOH, and –OH. They demonstrated that contact angles of these surfaces were 107°, 43°, 28°, and 25°, respectively. Moreover, the authors demonstrated that functionalized surfaces could adsorb increased amounts of fibronectin in the following trend: NH_2_ > CH_3_ > COOH > OH. Meanwhile, cell culture experiments showed that mouse preosteoblasts (MC3T3-E1 cell line) preferentially adhered to surface modified with OH groups, followed by COOH = NH_2_, while these cells did not attach to substrate modified with CH_3_ groups. In another study performed by Keselowsky et al. [173], it was found that surfaces functionalized with –OH and –NH_2_ groups significantly increased osteogenic differentiation of MC3T3-E1 cells compared to –COOH and –CH_3_ groups. According to the authors, the cell–biomaterial interactions do not depend on the amount of adsorbed proteins on the biomaterial surface but on their proper conformation. Likewise, Lee et al. [175] showed that SAM functionalized with -OH groups possessed the highest ability to adsorb fibronectin, while the surface with –NH_2_ groups possessed the best capacity to support adhesion of erythroleukemia cells (K562 cell line). Given these results, it is worth underlining that the presence of –NH_2_ (positive, hydrophilic) and –OH (neutral, hydrophilic) groups on biomaterial surface is more beneficial for cell adhesion, proliferation, and differentiation compared to –COOH (negative, hydrophilic) and –CH_3_ (neutral, hydrophobic) ones.

Combination of proteins/peptides with polymer scaffold can lead to introduce additional functional groups (Figure 2C). Aguirre-Chagala et al. [176] fabricated PCL nanofibers modified with collagen and elastin. They demonstrated that addition of these proteins significantly increased amount of –NH_2_ groups within fibrous matrix. Thus, the ninhydrin test revealed that the amount of amino groups within PCL (100 wt.%) and PCL/collagen/elastin (64:18:18 wt.%) fibers was equal to 0.36 and 21.0 mM NH_2_/mg fiber, respectively. Moreover, the measurement of contact angle indicated that surface of PCL/collagen/elastin composite possessed significantly higher wettability (θ=17°) compared to surface of PCL biomaterial (θ = 80°). Unfortunately, the authors did not evaluate biocompatibility of these fibers. In turn, Hu et al. [177] fabricated carboxymethyl chitosan sulfate (CMCS) modified with collagen peptide (COP) and proved that addition of COP to CMCS caused an increase in the number of –NH_2_ and –OH groups in the composite biomaterial. Moreover, they demonstrated that CMCS-COP significantly increased fibroblast viability compared to control. On the other hand, it is worth underlining that the functional groups of proteins/peptides and other polymers can react chemically with each other. For example, Socrates et al. [178] demonstrated that combination of collagen with chitosan led to loss of free hydrophilic groups (mainly –NH_2_ and –OH). The authors also showed that a lower number of hydrophilic groups resulted in a decreased surface wettability.

Taking into account the results obtained by different authors, it is difficult to establish a universal principle describing the influence of proteins/peptides addition on presence of functional groups in polymer-based biomaterials. However, inclusion of proteins and peptides that contain hydrophilic groups (mainly –NH_2_) usually increases the wettability and biocompatibility of biomaterial.

### 3.4. Surface Stiffness

Although wettability and chemistry of biomaterial surface are pondered as the most important properties which affect biocompatibility, it is worth underlining that surface stiffness (mainly expressed via Young’s modulus (E)) also plays an important role in the cell–biomaterial interactions [138,159,167,179,180]. It was found that surface stiffness regulates cell adhesion, spreading, migration, and proliferation. Moreover, surface rigidity also dictates stem cell fate. In other words, surface stiffness acts as physical signal that allows them to differentiate towards specific cells [167,179,181,182,183,184,185,186,187,188]. For instance, soft collagen-coated polyacrylamide gel (E = 1 kPa) did not support cell viability, spreading, or formation of the actin cytoskeleton, while the stiffer surface of such biomaterial (E equal to 8 kPa) enhanced adhesion and spreading of cells (studies on rat vascular smooth muscle cell line) [167,187]. On the other hand, the same polyacrylamide-based gels, which were characterized by different Young’s moduli, led to differentiation of MSCs towards following phenotypes: neuronal (E = 0.1–1 kPa), myogenic (E = 8–17 kPa), and osteogenic (E = 25–40 kPa) [167,188].

Inclusion of proteins/peptides to polymer scaffolds allows regulating their stiffness in order to obtain an appropriate template for tissue engineering applications (Figure 2D). Indeed, Jiang et al. [189] fabricated several scaffolds with different PCL/gelatin ratio—70:30 wt.%, 50:50 wt.%, or 30:70 wt.%—and they proved that biomaterial stiffness changed in a concentration-dependent manner. Among tested scaffolds, the biomaterial consisting of 70 wt.% PCL/30 wt.% gelatin possessed the highest stiffness, while the 30 wt.% PCL/70 wt.% gelatin biomaterial exhibited the lowest rigidity. Importantly, the surface of the 70 wt.% PCL/30 wt.% biomaterial was the most favorable for human BMSC adhesion, spreading, and proliferation. Hence, this research may corroborate that stiffer surface instead of softer substrate is more appropriate for cellular activities. Nevertheless, biomaterials characterized by lower stiffness are especially promising for soft tissue engineering applications (e.g., cardiovascular system or skin). Ryan and O’Brien [190] demonstrated that addition of insoluble elastin to collagen scaffold significantly reduced biomaterial stiffness and improved its viscoelastic properties. Cell culture experiments revealed that unlike collagen biomaterial, collagen/elastin scaffold promoted smooth muscle cell (SMC) differentiation towards contractile state as proven by enhanced expression of characteristic proteins—α-SMA, calponin, and SM-MHC. Similarly, Nguyen et al. [191] proved that Young’s moduli of collagen fibers enriched with insoluble or soluble elastin were approximately three- and five-fold lower compared to collagen fibers alone. However, both types of collagen/elastin fibers significantly increased expression of α-SMA, calponin, and thrombospondin in SMCs. Regarding mechanical and biological results, these collagen/elastin biomaterials are considered as promising candidates for cardiovascular tissue engineering application. Interestingly, Vázquez et al. [192] fabricated several PLGA/gelatin wound dressings and they demonstrated that PLGA/gelatin ratio had significant influence on stiffness and biocompatibility of these biomaterials. Thus, Young’s moduli were as follows: 72 ± 10 MPa (pure PCL), 48 ± 6 MPa (90 wt.% PLGA/10 wt.% gelatin), 58 ± 6 MPa (70 wt.% PLGA/30 wt.% gelatin), and 6 ± 1 MPa (50 wt.% PLGA/50 wt.% gelatin). Scanning electron microscope (SEM) observation revealed that the amount of MSCs grown on 70 wt.% PLGA/30 wt.% gelatin and 50 wt.% PLGA/50 wt.% gelatin was significantly higher compared to the amount of cells cultured on pure PCL as well as 90 wt.% PLGA/10 wt.% gelatin. Moreover, cells cultured on 70 wt.% PLGA/30 wt.% gelatin and 50 wt.% PLGA/50 wt.% gelatin were flattened and well spread, which suggested their good adhesion and growth. Mousavi et al. [39] proved that inclusion of collagen or gelatin to chitosan-based hydrogel significantly decreased its stiffness. The Young’s moduli of chitosan alone, chitosan/collagen, and chitosan/gelatin biomaterials were close to 154, 67, and 52 kPa, respectively. In turn, the number of fibroblasts cultured on chitosan/collagen and chitosan/gelatin biomaterials was 1.3- and 2-fold higher in comparison with the number of cells grown on chitosan hydrogel. The authors suggested that the best proliferation of fibroblasts on chitosan/gelatin hydrogel may result from higher amount of RGD sequences and a lower surface stiffness compared to either chitosan or chitosan/collagen biomaterials.

### 3.5. Degradability

It is widely known that biomaterials for tissue engineering applications should be not only biocompatible but also should have the ability to degrade. A biomaterial’s capacity to degrade as well as its degradation rate depends on many features, such as chemical structure, occurrence of hydrolytically unstable bonds, hydrophilicity/hydrophobicity, and molecular weight [9,10,13,19]. Degradation of biomaterials takes place via physicochemical and/or biological processes. These events lead to cleavage of hydrolytically or enzymatically sensitive bonds in the scaffolds. Importantly, degradation rate of biomaterials should be correlated with the time needed to form ECM by the cells. Moreover, the products that arise during degradation should be non-toxic towards surrounding tissues and should exist in the body without adverse effects [9,18,19,193,194].

In general, proteins and peptides degrade too fast, but their degradation products are usually non-toxic. In the case of other polymers, especially synthetic ones, the degradation rate is slower, but by-products of degradation (inter alia acidic by-products) may lead to inflammation or necrosis of cells and tissues. To overcome these drawbacks, proteins and peptides are combined with polymers scaffolds [9,10,19,195,196]. Such association allows to obtain biomaterials with optimal rate of degradation (Figure 2E). For instance, Mousavi et al. [39] compared degradation ability of chitosan, chitosan/collagen, and chitosan/gelatin biomaterials. They proved that after 15-day incubation in phosphate buffered saline (PBS) with lysozyme, the mass loss of biomaterials increased in the following trend: chitosan/gelatin > chitosan/collagen > chitosan. The authors suggested that the greatest degradation rate of chitosan/gelatin biomaterial can be associated with its large hydrophilic nature as it also exhibited the highest ability to absorb liquid compared to other scaffolds. Badhe et al. [197] fabricated bi-layered tubular chitosan/gelatin scaffold. They demonstrated that composite scaffold possessed intermediate ability to degrade in comparison with chitosan and gelatin biomaterials (tested using PBS–lysozyme solution). The gelatin alone exhibited approximately 93% mass loss within one day of experiment. In turn, after 30-day incubation, the mass loss of chitosan/gelatin and chitosan biomaterials was 78.0% ± 5.0% and 64.0% ± 8.0%, respectively. It was justified, because gelatin alone possessed significantly higher ability to absorb PBS compared to chitosan and chitosan/gelatin scaffolds. In turn, Prado-Prone et al. [163] fabricated PCL/gelatin scaffolds and demonstrated that addition of higher amount of gelatin to polymer scaffold resulted in faster degradation. After three-day incubation in PBS, the mass loss of PCL alone, 70 wt.% PCL/30 wt.% gelatin, and 55 wt.% PCL/45 wt.% gelatin was close to 4%, 26%, and 43%, respectively. Likewise, Gil-Castell et al. [198] clearly confirmed that degradation rate of PCL-based biomaterials tightly depended on amount of incorporated gelatin. They noted that, after 60-day incubation in PBS, the mass loss of PCL was approximately 40%. In the case of 50 wt.% PCL/50 wt.% gelatin and 40 wt.% PCL/60 wt.% gelatin, the mass loss was close to 60% and 80%, respectively. Importantly, the authors also evaluated in vitro and in vivo inflammatory response induced by PCL alone as well as 40 wt.% PCL/60 wt.% gelatin biomaterial. The pyrogen test demonstrated that PCL scaffold did not stimulate pro-inflammatory cytokines (IL-1β, IL-6, IL-10, and TNF-α) production by peripheral blood mononuclear cells (PBMNCs). Upon incubation of cells with the 40 wt.% PCL/60 wt.% gelatin biomaterial, the level of pro-inflammatory cytokines was similar to the level obtained with positive control (polyhydroxybutyrate (PHB)). Although in vitro assay showed an increase in pro-inflammatory cytokine production by the cells incubated with 40 wt.% PCL/60 wt.% gelatin biomaterial, the in vivo assessment did not confirm these results. Thus, after 15 days of subcutaneous implantation of PCL and 40 wt.% PCL/60 wt.% gelatin, no chronic effect was observed (studies on C57B6J mice).

## 4. Application of Polymer Scaffolds Modified with Proteins and Peptides

Biomaterials for tissue engineering applications have to meet numerous requirements. They should be primarily biocompatible, biodegradable, and mechanically robust. Moreover, they should constitute 3D interconnected porous constructs that mimic structure of natural ECM. Nevertheless, the specified features of biomaterials (e.g., pore size or mechanical strength) are closely associated with their future applications. For instance, biomaterials for skin TE may possess lower mechanical strength compared to scaffolds dedicated for bone TE [9,10,11,13,17,18,31,33,196,199,200,201].

In recent years, biocompatible polymer scaffolds modified with proteins/peptides have been developed for regeneration of skin [192,202,203], nerves [204,205,206], muscles [207,208], tendons [209,210,211], cartilages [212,213,214], and bones [57,58,215]. To fabricate these biomaterials, many different techniques are applied. Thus, porous scaffolds can be fabricated using freeze-drying, solvent casting/particulate leaching, or 3D printing, while fibrous biomaterials can be produced via electrospinning, self-assembly, phase separation, and solid free-form fabrication [4,9,17,19,200,216]. However, based on recent data, it seems that freeze-drying [57,217,218,219] and electrospinning [58,192,207,215,220] are most frequently used. Freeze-drying, also referred to as lyophilization, is a simple method that allows obtaining 3D porous scaffolds. In this method, polymer mixture or hydrogel (if it was cross-linked before) are firstly frozen (e.g., at −20 °C or −80 °C), which enables formation of construct with numerous ice crystals. Then, such frozen product is allocated to consequent drying in a freeze-dryer, in which ice crystals are removed via sublimation. The empty areas, previously occupied by ice crystals, become the pores within biomaterial structure. The main advantages of freeze-drying are the ability to regulate pore size from 20 to approximately 300 μm by the change of freezing procedure as well as the possibility to obviate high temperature. In turn, long timescale, high energy consumption, and the possibility to generate irregular pore size constitute the drawbacks of this method [123,221,222,223,224]. Electrospinning is more complicated technique in which electrostatic forces are used for formation of thin fibers from polymer solution. Briefly, in this method, the electrically charged jet of polymer solution is formed by the high voltage. On the way to the collector, the discharged polymer solution undergoes an instability process, which results in elongation of the jet and evaporation or solidification of the solvent. Finally, the interconnected web of ultrathin fibers is collected. In general, fabricated nano- or microfibers (range from 10 nm to 100 μm) possess controllable morphology. Electrospinning requires the presence of specialized equipment including syringe pump, metallic needle, high-voltage supply, and grounded collector. Moreover, the problem of obtaining a 3D structure with suitable pore size is considered as a main disadvantage of this technique. Importantly, electrospinning does not require the use of high temperature [19,196,216,221,225]. It is worth underlining that fabrication of proteins-based biomaterials should be performed under mild conditions. The use of high temperature (>40 °C), organic solvents, or strongly acidic/alkaline solutions leads to destroying the structural integrity and loss of biological activity of these biomolecules [226,227]. Thus, the choice of adequate conditions during fabrication process of proteins-based biomaterials need to considered. All the above-mentioned techniques have been described and discussed in detail by many researchers. Thus, well-structured knowledge about these methods and their advantages/limitations can be found in more specialized papers [196,200,216,220,221,222,228,229,230,231].

### 4.1. Skin TE

Skin is the largest organ of the human body, consisting of epidermis, dermis, and hypodermis. It constitutes a protective barrier towards outer world, and thus is susceptible to damage resulting from inter alia burns, surgical procedures, or diabetic ulcers. To enhance wound repair and regeneration, many tissue-engineered skin products have been developed [232,233,234,235,236]. In general, biomaterials for treatment of skin injuries can be divided on two groups including wound dressings and skin substitutes (Table 1). 

**Figure 3:**
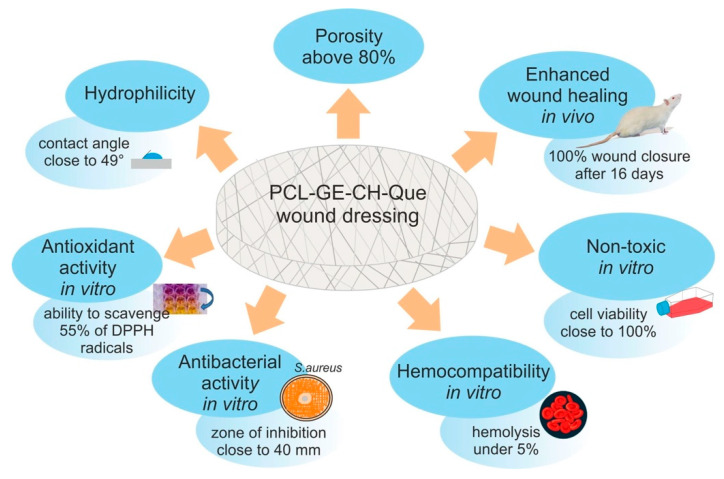
Schematic illustration presenting main properties of PCL-GE-CH-Que wound dressing fabricated by Ajmal et al. [247]. Abbreviations: CH, ciprofloxacin hydrochloride; DPPH, 2,2-diphenyl-1-picrylhydrazyl; GE, gelatin; PCL, poly-ε-caprolactone; Que, quercetin. Figure 3 in this review was prepared by the authors yourself, based on results described in [247].

Wound dressings constitute a barrier that protects skin from harmful factors of external environment as well as microbial infections. The appropriate wound dressings should be non-toxic and they should maintain suitable wound environment. In other words, they should have optimal water vapor transmission ratio (WVTR) and great ability to absorb wound exudate (if it occurs). Moreover, modern wound dressings should also accelerate wound healing [232,233,234,236,242,243,244,245]. Progress in the field of engineering of biomaterials has led to the development of many promising wound dressings. Mousavi et al. [39] fabricated collagen/chitosan and gelatin/chitosan scaffolds using freeze-drying technique. They demonstrated that both biomaterials were non-toxic towards fibroblast cells. Nevertheless, gelatin/chitosan scaffold possessed slightly higher liquid uptake ability (swelling ratio close to 30%) compared to collagen/chitosan dressing (swelling ratio equal to 20%). Moreover, unlike gelatin/chitosan biomaterial, collagen/chitosan scaffold allowed for optimal water vapor transmission (WVTR of approximately 2750 g/m^2^/day). Collagen/chitosan biomaterial also possessed slightly higher mechanical properties and significantly slower biodegradation rate in comparison with gelatin/chitosan scaffold. Thus, the authors suggested that collagen/chitosan biomaterial could be a promising candidate for wound dressing. Akhavan-Kharazian and Izadi-Vasafi [246] fabricated chitosan/gelatin/nanocrystalline cellulose/calcium peroxide wound dressing. They demonstrated that it possessed excellent liquid uptake ability (swelling ratio around 2000%) and great mechanical properties (E = 2262 ± 110 MPa). Moreover, this dressing allowed for water vapor transmission (WVTR equal to 40 g/m^2^/h) and it possessed antibacterial activity. After 24-h incubation, the zones of inhibition for *E. coli* and *S. aureus* were 22 and 16 mm, respectively. Meanwhile, cell culture experiments revealed that this biomaterial did not exhibit cytotoxic effect towards mouse fibroblasts (NIH/3T3 cell line). Importantly, wound dressings may be also modified with natural and/or synthetic compounds in order to improve their antioxidant, anti-inflammatory, and antibacterial properties [238,247]. For instance, Ajmal et al. [247] fabricated electrospun gelatin/PCL wound dressing modified with ciprofloxacin hydrochloride (CH) and quercetin (Que). The authors demonstrated that resultant PCL-GE-CH-Que dressing possessed antioxidant and antibacterial properties in vitro. Moreover, this biomaterial was non-toxic in vitro (studies on 3T6-Swiss albino fibroblasts), and it accelerated wound healing in Wistar rats (Figure 3).

On the other hand, serious skin injuries, such as deep dermal and full thickness wounds, require complex treatment using skin substitutes. Skin substitutes should not only protect wound from detrimental factors, but they should primarily replace the functions of skin (temporarily or permanently). Thus, they should be characterized by flexibility as well as lack of toxicity and antigenicity. Moreover, such biomaterials should promote cell migration, proliferation, and differentiation, as well as accelerate re-epithelialization and neovascularization. Skin substitutes should also possess mechanical parameters close to those of natural skin (inter alia Young’s modulus. ranging 60–850 kPa) [232,233,234,236,242,243,244,245,248]. Nowadays, there is no biomaterial that meets all these criteria. Development of an “ideal” skin substitute remains a big challenge for researchers. Chong et al. [202] produced electrospun collagen/elastin/PCL scaffold (CEP 1) and demonstrated its potential as a dermal substitute. The authors proved that, thanks to the presence of elastin, the obtained biomaterial possessed optimal elasticity (tensile modulus close to 108 kPa). Moreover, the resultant scaffold promoted adhesion, growth, and proliferation of fibroblasts and keratinocytes in vitro (studies on HDF and HaCaT cell lines). Importantly, the CEP 1 biomaterial significantly accelerated tissue integration and early-stage angiogenesis compared to commercial skin substitute, Integra^®^ (studies on Balb/c mice). Chen et al. [249] produced chitosan hydrogel enriched with SIKVAV (Ser-Ile-Lys-Val-Ala-Val) sequence derived from A chain of laminin. They proved that SIKVAV/chitosan hydrogel promoted wound healing, re-epithelialization, angiogenesis, proliferation, and differentiation of keratinocytes. Moreover, it inhibited inflammation in skin wounds (studies on C57BL/mice). You et al. [250] fabricated collagen/chitosan scaffold modified with silver nanoparticles (NAg). The in vitro experiments showed that NAg/collagen/chitosan biomaterial possessed antibacterial activity against *E. coli* and *S. aureus* as well as promoted migration of skin cells (studies on co-culture of mouse embryo fibroblasts and human skin keratinocytes (HaCaT)). Moreover, in vivo studies demonstrated that this biomaterial supported skin repair and regeneration. Sixty days after transplantation of NAg/collagen/chitosan biomaterial into skin wounds of Sprague-Dawley rats, their regenerated skin possessed similar structure to normal skin.

### 4.2. Nerve TE

The nervous system involves the central nervous system (CNS) and the peripheral nervous system (PNS). The brain and spinal cord make up the CNS. In turn, the PNS is composed of the ganglia and nervous tissue outside of the CNS. Because nervous tissue exhibits very poor ability to regenerate, damaged components of nervous system require adequate therapeutic interventions. Treatment of CNS injuries mainly focuses on preventing any further damage, while treatment of PNS injuries primarily involves the use of nerve autografts. Although nerve autografts are considered as a “gold standard”, their application possesses many limitations. Thus, there is an increasing trend towards design of tissue-engineered nerve grafts [242,243,251,252,253]. The biomaterials for treatment of PNS injuries should mainly promote the viability and growth of Schwann cells, because they are crucial for myelination of axons. Thus, such scaffolds should be biocompatible and porous (porosity higher than 50% allows to supply nutrients and oxygen to the cells). Importantly, biomaterials with pore size ranging 30–50 μm allow for survival and migration of Schwann cells. The pore size of scaffolds dedicated for regeneration of long-size peripheral axons should range 200–750 μm. Furthermore, these biomaterials should be biodegradable and they should possess appropriate mechanical properties close to those of human nerves (inter alia Young’s modulus ranging 5–16 MPa) [243,245,251,252,253,254,255].

The current trend in the field of engineering of biomaterials for peripheral nerve repair primarily focuses on nanofibers. The combination of polymer nanofibers with proteins such as collagen, gelatin, or laminins allows improving the response of Schwann cells as well as enhancing outgrowth of nerve axons and nerve functional recovery. Thus, such nanofibers are most often used for the fabrication of nerve guidance conduits (NGCs). NGCs act as fillers of the nerve gaps between broken ends, and they support outgrowth of the nerve itself [18,232,252,254,256].

The NGCs’ ability to support cellular activity has been revealed during in vitro studies. For instance, Singh et al. [204] fabricated nanofibrous gelatin/chitosan/polyurethane nerve conduit with pore diameter of 29.60 ± 9.83 μm. They demonstrated that such biomaterial was non-toxic towards BMSCs as well as Neuro 2 cells (mouse neuroblastoma cell line). Moreover, it enhanced adhesion, growth, and proliferation of these cells. KarbalaeiMahdi et al. [257] produced gelatin/PCL nanofibers and they showed that their porosity was above 50%. The authors also proved that these biomaterials possessed great mechanical properties (E close to 100 MPa). Importantly, gelatin/PCL nanofibers exhibited ability to enhance neural differentiation of hiPSCs (human induced pluripotent stem cells). Silantyeva et al. [205] fabricated PCL nanofibers functionalized with GYIGSR (Gly-Tyr-Ile-Gly-Ser-Arg) sequence derived from B1 chain of laminin. They showed that such biomaterials promoted neural differentiation of mouse embryonic stem cells (ES-D3 cell line). Song et al. [258] produced electrospun polydioxanone/collagen (PDO/Col) and laminin/polydioxanone/collagen (Lam-PDO/Col) core–shell matrices. They demonstrated that Lam-PDO/Col biomaterial possessed interconnected porous structure and moderate mechanical properties (E close to 2.5 MPa). Moreover, it provided sustained release of laminin up to 28 days. Cell culture experiments revealed that the number of cells grown on Lam-PDO/Col biomaterial was significantly higher compared to the number of cells cultured on PDO/Col scaffold (studies on hippocampal neuronal cells—HT-22 cell line). The HT-22 cells seeded on Lam-PDO/Col biomaterial possessed extensive system of cytoskeletal filaments, which proved their good growth and proliferation. The authors suggested that this phenomenon results from presence of IKVAV sequence in laminin, which possesses the ability to promote adhesion and proliferation of neuronal cells.

**Figure 4:**
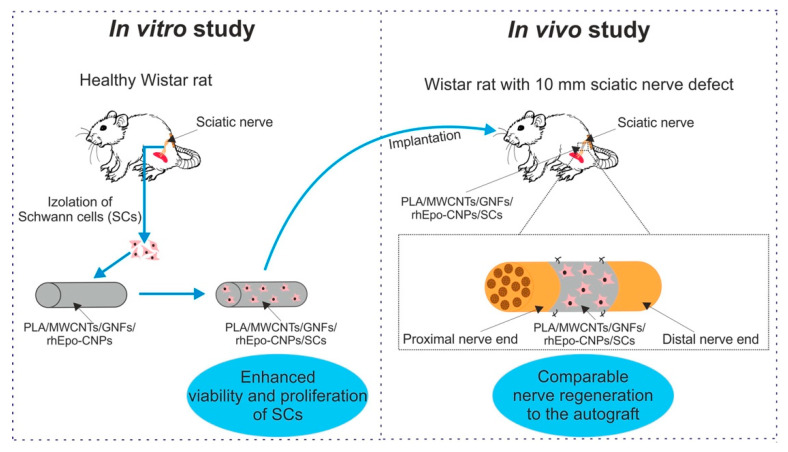
Schematic illustration presenting in vitro and in vivo studies of PLA/MWCNTs/GNFs/rhEpo-CNPs nerve conduit performed by Salehi et al. [219]. Abbreviations: GNFs, gelatin nanofibrils; MWCNTs, multiwalled carbon nanotubes; PLA, polylactic acid; rhEpo-CNPs, recombinant human erythropoietin-loaded chitosan nanoparticles; SCs, Schwann cells. Figure 4 in this review was prepared by the authors yourself, based on results described in [219].

The complex evaluation of NGCs ability to support peripheral nerve repair should include either in vitro and in vivo studies. It is worth noting that the choice of appropriate animal models is crucial to obtain reliable results. In other words, some of them are not adequate for imitation the conditions in human body. For instance, it was proved that nerve axons in mice are smaller and shorter compared to axons in humans. Moreover, there is a significant difference between damage recovery in mice and humans. For these reasons, in vivo studies on rats and larger animals are necessary to evaluate potential of new biomaterials for nerve TE applications [18,254,256]. Chang et al. [206] fabricated nanofibrous PCL as well as PCL/laminin nerve conduits and demonstrated that pore size of both biomaterials was close to 150 μm. Cell culture experiments showed that viability of cells grown on PCL/laminin scaffold was significantly higher compared to cells cultured on PCL biomaterial (studies on PC-12 cell line). Moreover, in vivo experiments revealed that PCL/laminin scaffold supported nerve regeneration (studies on Sprague Dawley rats). Li et al. [259] demonstrated that laminin/chitosan/PLGA nerve conduit combined with Schwann cells and neural stem cells promoted nerve regeneration in vivo (studies on Sprague Dawley rats). Interestingly, Salehi et al. [219] fabricated biodegradable nerve conduit consisting of PLA, multi-walled carbon nanotubes (MWCNTs), and gelatin nanofibrils (GNFs) combined with the recombinant human erythropoietin-loaded chitosan nanoparticles (rhEpo-CNPs). This biomaterial possessed porous structure (porosity above 85%), and it released rhEpo in a sustained manner up to 14 days. Mechanical test demonstrated that such conduit possessed higher tensile strength compared to fresh transected adult rat sciatic nerve. Moreover, the MTT and LDH assays revealed that PLA/MWCNTs/GNFs/rhEpo-CNPs conduit promoted viability and proliferation of Schwann cells, while in vivo studies demonstrated that it enhanced regeneration of the sciatic nerve in Wistar rats (Figure 4).

### 4.3. Bone TE

Bone is a highly specialized tissue that possesses the ability to regenerate. However, traumatic injuries and serious diseases (e.g., osteoporosis or osteoarthritis) result in significant decrease of bone regeneration capacity. Bone is mainly composed of calcium phosphate (i.e., hydroxyapatite (HAp)) and collagen Type I. The outer layer of bone (cortical bone) possesses compact structure, with porosity ranging 5%–20%. The Young’ modulus of cortical bone is between 10 and 20 GPa. The inner layer of bone (cancellous bone) has extremely porous structure (porosity between 50% and 90%) with pore size ranging 300–600 μm. In comparison with cortical bone, cancellous bone exhibits lower mechanical parameters with Young’s modulus of 20–500 MPa [3,72,245,260,261,262].

Ideally, biomaterials for bone TE applications should be biocompatible, bioactive, osteoconductive, osteoinductive, and biodegradable at controllable rate (even up to nine months). Moreover, they should possess 3D interconnected porous structure as well as exhibit good mechanical properties. Pore interconnectivity enables adequate penetration of nutrients to cells within the scaffolds and it allows for diffusion of waste products out of the biomaterials. Moreover, the pore size affects osteoconductive and osteoinductive properties of the scaffolds. Pore sizes ranging 100–350 μm are suitable for osteoblast growth, proliferation, and differentiation. A pore size above 300 μm enables angiogenesis and bone ingrowth into implanted biomaterial. Nevertheless, bone scaffolds with high porosity usually possess low mechanical properties. Thus, fabrication of extremely porous and mechanically robust biomaterials is still problematic [8,243,260,261]. 

In recent years, many polymer scaffolds modified with proteins/peptides for bone TE applications have been developed (Table 2). Importantly, they can be combined with inorganic components, such as synthetic HAp, alpha- and beta-tricalcium phosphate (α- and β-TCP) as well as bioactive glass in order to improve their structural similarity to natural bone, mechanical properties, and osteoconductivity [72,256,260,261,262,263].

For instance, Janarthanan et al. [273] fabricated PCL/α-TCP, gelatin/PCL/α-TCP, and fibronectin/PCL/α-TCP scaffolds using solvent casting method combined with gas foaming process. They showed that both gelatin/PCL/α-TCP and fibronectin/PCL/α-TCP scaffolds were characterized by superior biocompatibility in vitro compared to PCL/α-TCP biomaterial (studies on human ASCs). Bhuiyan et al. [274] fabricated collagen/PLGA/nHAp biomaterial using ring-opening polymerization technique and demonstrated that it exhibited mechanical properties close to those of human cancellous bone (E was approximately 60 MPa). Moreover, cell culture experiments showed that this scaffold promoted proliferation and osteogenic differentiation of human BMSCs. Maji et al. [275] fabricated gelatin/carboxylmethyl chitosan/nHAp (SGC) scaffold using freeze-drying technique. The resultant SGC biomaterial possessed macroporous structure (porosity above 90%) with pore size close to 600 μm. It also exhibited controllable degradation rate during incubation in PBS solution containing collagenase Type I and lysozyme. Importantly, SGC scaffold was biocompatible and possessed osteoinductive properties in vitro, as it enhanced proliferation and osteogenic differentiation of wjhMSC (human Wharton’s jelly-derived mesenchymal stem cells). Nevertheless, this biomaterial exhibited relatively low mechanical properties (E equal to 12.28 MPa). Thus, it seems that SGC biomaterial should be used for non-load bearing implantation sites.

**Figure 5:**
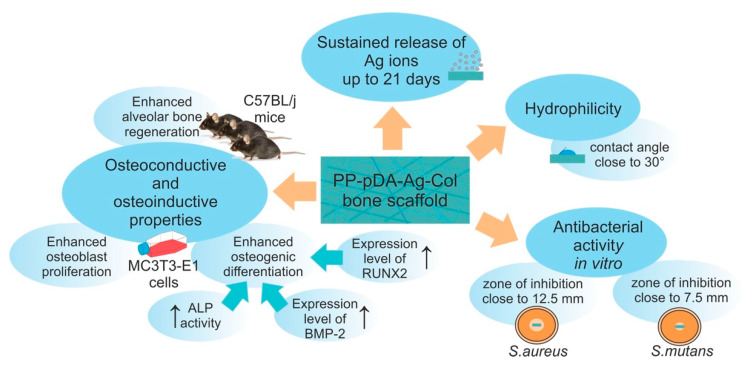
Schematic illustration presenting main properties of PP-pDA-Ag-Col bone scaffold fabricated by Qian et al. [280]. Abbreviations: ALP, alkaline phosphatase; BMP-2, bone morphogenetic protein-2; Col, collagen; pDA, polydopamine; PP, poly(lactic-co-glycolic acid)/poly-ε-caprolactone. Figure 5 in this review was prepared by the authors yourself, based on results described in [280].

It is worth emphasizing that implantable scaffolds are compromised by bacterial infections. The current strategy for the prevention of biomaterial-associated infection (BAI) mainly involves long-term systemic administration of high doses of antibiotics. Unfortunately, such therapy is expensive and may lead to systemic toxicity in patients. Modification of bone scaffolds with antibacterial agents such as antibiotics, antibacterial peptides, and metal nanoparticles (e.g., AgNPs, CuNPs, or ZnNPs) constitutes promising protection strategy against BAI. However, it should be taken into account that incorporated antibacterial agents not only inhibit growth of bacterial cells, but they also may exhibit toxic effect towards eukaryotic ones. The combination of polymer scaffolds with biocompatible proteins/peptides and antibacterial agents allows keeping a balance between suitable antibacterial protection and cytotoxicity [276,277,278,279,280,281]. For instance, Mantripragada and Jayasuriya [279] fabricated chitosan microparticles enriched with BMP-7 and antibiotics, i.e., vancomycin and cefazolin. They demonstrated that both types of microparticles—BMP-7/chitosan/vancomycin and BMP-7/chitosan/cefazolin—exhibited antibacterial activity against *S. epidermidis*. Fluorescent microscope observation revealed that addition of BMP-7 to chitosan/vancomycin and chitosan/cefazolin biomaterials significantly enhanced viability of mouse preosteoblasts (OB-6 cell line). Qian et al. [280] fabricated electrospun PLGA/PCL bone scaffold modified with collagen, polydopamine, and AgNPs. The authors demonstrated that resultant PP-pDA-Ag-Col biomaterial as well as control scaffold (PP-pDA-Ag) possessed comparable antibacterial activity against *S. aureus* and *S. mutans*. Cell culture experiments showed that PP-pDA-Ag-Col scaffold significantly enhanced adhesion, proliferation, and osteogenic differentiation of mouse preosteoblasts (MC3T3-E1 cells) in comparison with PP-pDA-Ag biomaterial. Moreover, in vivo studies on C57BL/j mice with periodontitis revealed that PP-pDA-Ag-Col scaffold promoted alveolar bone regeneration. The main properties of this bone scaffold are presented in Figure 5.

## 5. Conclusions and Future Perspectives

There is still an increasing trend towards designing biocompatible biomaterials for tissue engineering applications. The biodegradable natural and synthetic polymer scaffolds constitute a promising approach in the field of engineering of biomaterials due to their structural and mechanical properties. Their framework and composition should mimic natural ECM, which not only regulates cellular processes but also makes up mechanical support for cell growth. Unfortunately, in some cases, biocompatibility of fabricated polymer scaffolds is insufficient to enable proper cell attachment, proliferation, and differentiation. Consequently, such biomaterials do not allow for fast tissue repair and regeneration. Modification of scaffolds, primarily with bioactive molecules, enhances their biological properties. Considering the composition of native tissues, ECM proteins, adhesive peptides, and growth factors are most often used for enrichment of polymer biomaterials. Remarkable progress in molecular biology and biotechnology has resulted in the discovery of novel proteins and peptides that can be used in the field of engineering of biomaterials. Nevertheless, manufacturing and purification process of such products are still extremely expensive. Moreover, synthesized proteins and peptides may induce immune response by body. For these reasons, there is a need to search new, biocompatible, non-immunogenic, and cost-effective proteins and peptides that may be used for tissue engineering applications.

It is worth underlining that current research trend in the field of engineering of biomaterials focuses on fabrication of smart multifunctional scaffolds that exhibit broad spectrum of activity. Thus, in vitro and in vivo studies have shown that combination of polymers, proteins/peptides, and active components (e.g., natural and synthetic compounds, metal nanoparticles, etc.) leads to obtaining biocompatible composite biomaterials, which enhance wound healing, re-epithelialization, and angiogenesis as well exhibit antibacterial properties. Although the number of articles presenting fabrication process and in vitro assessment of such biomaterials is increasing from year to year, there are still too few in vivo studies and clinical data that would confirm their effectiveness. Thus, newly fabricated scaffolds should be allocated to complex evaluation in vitro, followed by in vivo studies and clinical trials.

## Figures and Tables

**Table 1:** Summary of some biodegradable polymer scaffolds modified with proteins/peptides for skin TE applications.

Biomaterial Composition	Fabrication Method	In Vitro/ In Vivo Experimental Models	Main Advantages	Application	Ref.
Collagen peptides/ carboxymethyl chitosan	Covalent coupling/ freeze-drying	L929 cell line/ Rabbits	Enhances cell viability and migration in vitro, promotes skin regeneration in vivo	Wound dressing	[237]
Gelatin/ chitosan	Cross-linking by tannin/ freeze-drying	L929 cell line/ Rabbits	Porous structure, good mechanical properties, good water absorption and retention capacities, non-toxic in vitro, possesses in vitro antibacterial activity against *S. aureus* and *E. coli*, enhances wound healing in vivo	Wound dressing	[217]
Gelatin/ chitosan/ lupeol	Solution casting	NIH/3T3 cell line	Non-brittle, flexible, suitable water vapor transmission, excellent swelling ability, possesses antioxidant activity in vitro, non-toxic in vitro, possesses in vitro antibacterial activity against *P. aeruginosa*	Wound dressing	[238]
Gelatin/ PLGA	Electrospinning	Human MSCs/ Rats	Hydrophilic surface, non-toxic in vitro, promotes cell proliferation in vitro, biodegradable at controllable rate, non-toxic in vivo	Wound dressing	[192]
Collagen/ hyaluronic acid	Blending	Mice	High complex viscosity, low weight change after injecting, enhances fibroblast migration in vivo, promotes vascularization in vivo	Skin substitute	[239]
Collagen/ hyaluronic acid-tyrosine	Crosslinking by BDDE	L929 cell line/ Rabbits	Good mechanical properties, biodegradable at controllable rate, supports cell viability in vitro, enhances cell adhesion in vitro, biocompatible in vivo	Skin substitute	[240]
Gelatin/ chitosan/ PCL/ curcumin	Electrospinning	Human EnSCs/Rats	Hydrophilic surface, porous structure, good mechanical properties, biodegradable at controllable rate, enables sustained-release of curcumin, non-toxic in vitro, promotes cell proliferation in vitro, enhances wound healing in vivo	Skin substitute	[241]
VEGF/ PLGA	Encapsulation via solvent evaporation technique	HaCaT and BJ cell lines/ Mice	Non-toxic in vitro, enhances cell migration and proliferation in vitro, enhances wound healing in vivo, promotes re-epithelialization and neovascularization in vivo	Skin substitute	[203]

**Table 2:** Summary of some biodegradable polymer scaffolds modified with proteins/peptides for bone TE applications.

Biomaterial Composition	Fabrication Method	In Vitro/ In Vivo Experimental Models	Main Advantages	Ref.
ELP/collagen	Blending	human ASCs	Non-toxic in vitro, promotes osteogenic differentiation in vitro;	[264]
Collagen/ chitosan	Electrospinning	Human PDLCs/ Rats	Highly porous structure, good mechanical properties, biodegradable at controllable rate, non-toxic in vitro, promotes osteogenesis in vivo	[58]
Gelatin/chitosan	Cross-linking by glutaraldehyde/ freeze-drying	Human DPSCs/ Mice	Porous structure, non-toxic in vitro, enhances cell proliferation in vitro, enhances bone regeneration in vivo	[265]
Collagen/ hyaluronic acid	Dip coating	Rabbit BMSCs	Non-toxic in vitro, promotes osteogenic differentiation in vitro	[266]
Gelatin/PLA/PCL/ metformin	Freeze casting technique	MG-63 cell line/ BMSCs/ Rats	Porous structure, good mechanical properties, non-toxic in vitro, promotes cell proliferation in vitro, promotes osteogenic differentiation in vitro, enhances bone regeneration in vivo	[267]
Cell-derived dECM/ PLGA/PLA	Freeze-drying	UCB-MSCs	Non-toxic in vitro, enhances cell growth and proliferation in vitro, promotes osteogenic differentiation in vitro	[112]
PLA/polydopamine/ BMP-2	3D printing	Rabbit BMSCs/Rats	Porous structure, hydrophilic surface, good mechanical properties, sustained release of BMP-2, promotes cell adhesion and proliferation in vitro, promotes osteogenic differentiation in vitro, enhances bone regeneration in vivo	[268]
Graphene oxide/HAp/PLGA/BMP-2	Emulsion-solvent evaporation	MC3T3-E1 cell line	Non-toxic in vitro, promotes cell adhesion and proliferation in vitro, promotes osteogenic differentiation in vitro, enhances calcium deposition in vitro	[269]
PCL/β-TCP/bdECM/ rhBMP-2	3D printing	MC3T3-E1 cell line/ Rats	Porous structure, sustained release of rhBMP-2, promotes cell adhesion and proliferation in vitro, promotes osteogenic differentiation in vitro, enhances bone regeneration in vivo	[270]
P15-OPG peptides/ pDA/op-HAp/PLGA	Freeze-drying	MC3T3-E1 cell line	Hydrophilic surface, non-toxic in vitro, promotes cell proliferation in vitro, promotes osteogenic differentiation in vitro	[166]
Gelatin/PLGA/HAp	Electrospinning	Human ADSCs	Interconnected porous structure, non-toxic in vitro, promotes cell adhesion and proliferation in vitro, promotes osteogenic differentiation in vitro	[215]
Gelatin/chitosan/ bioactive glass	Blending	Rat BMSCs/ Swiss rats	Bioactive in vitro, non-toxic in vitro, enhances bone regeneration in vivo	[271]
Collagen/ functionalized multiwalled carbon nanotube/chitosan/ HAp	Freeze-drying	MG-63 cell line	Interconnected porous structure, non-toxic in vitro, bioactive in vitro	[57]
Collagen/ chitosan/PCL/ graphene oxide	Electrospinning	MG-63 cell line	Hydrophilic surface, bioactive in vitro, promotes cell adhesion, proliferation, and osteogenic differentiation in vitro	[272]

## Abbreviations

3D	Three-dimensional
AgNPs = NAg	Silver nanoparticles
ALP	Alkaline phosphatase
ASCs	Adipose tissue-derived stem cells
BAI	Biomaterial-associated infection
BCP	Biphasic calcium phosphate
BDDE	1,4-butanedioldiglycidyl ether
bdECM	Bone demineralized extracellular matrix
BMPs	Bone morphogenetic proteins
BMSCs	Bone marrow-derived stem cells
BSP	Bone sialoprotein
CH	Ciprofloxacin hydrochloride
CMCS	Carboxymethyl chitosan sulfate
CNS	Central nervous system
COP	Collagen peptide
CuNPs	Copper nanoparticles
dECM	Decullarized extracellular matix
DGEA	Asp-Gly-Glu-Ala amino acid sequence
DPPH	2,2-diphenyl-1-picrylhydrazyl
DPSCs	Dental pulp stem cells
E	Young’s modulus (Modulus of elasticity)
ECM	Extracellular matrix
ELPs	Elastin-like polypeptides
ELRs	Elastin-like recombinamers
EnSCs	Endometrial stem cells
FBS	Fetal bovine serum
FGF-2	Basic fibroblast growth factor-2
GAGs	Glycosaminoglycans
GE	Gelatin
GFOGER	Gly-Phe-HPro-Gly-Glu-Arg amino acid sequence
GFPGER	Gly-Phe-Pro-Gly-Glu-Arg amino acid sequence
GFs	Growth factors
GNFs	Gelatin nanofibrils
GQGFSYPYKAVFSTQ	Gly-Gln-Gly-Phe-Ser-Tyr-Pro-Tyr-Lys-Ala-Val-Phe-Ser-Thr-Gln amino acid sequence
GTPGPQGIAGQRGVV	Gly-Thr-Pro-Gly-Pro-Gln-Gly-Ile-Ala-Gly-Gln-Arg-Gly-Val-Val amino acid sequence
GYIGSR	Gly-Tyr-Ile-Gly-Ser-Arg amino acid sequence
HA	Hyaluronic acid
HAp	Hydroxyapatite
IGF-1	Insulin-like growth factor-1
IKVAV	Ile-Lys-Val-Ala-Val amino acid sequence
IS	Insoluble elastin
IVAPPGYHAFYCHGECP	Ile-Val-Ala-Pro-Pro-Gly-Tyr-His-Ala-Phe-Tyr-Cys-His-Gly-Glu-Cys-Pro amino acid sequence
KAFDITYVRLKF	Lys-Ala-Phe-Asp-Ile-Thr-Tyr-Val-Arg-Leu-Lys-Phe amino acid sequence
KIPKASSVPTELSAISTLYLSGGC	Lys-Ile-Pro-Lys-Ala-Ser-Ser-Val-Pro-Thr-Glu-Leu-Ser-Ala-Ile-Ser-Thr-Leu-Tyr-Leu-Ser-Gly-Gly-Cys amino acid sequence
KLTWQELYQLKYKGI	Lys-Leu-Thr-Trp-Gln-Glu-Leu-Tyr-Gln-Leu-Lys-Tyr-Lys-Gly-Ile amino acid sequence
KQAGDV	Lys-Gln-Ala-Gly-Asp-Val amino acid sequence
KQLNAISVLYFDD	Lys-Gln-Leu-Asn-Ala-Ile-Ser-Val-Leu-Gln-Phe-Asp-Asp amino acid sequence
LDV	Leu-Asp-Val amino acid sequence
MB	Basement membrane of extracellular matrix
MSCs	Mesenchymal stem cells
MWCNTs	Multi-walled carbon nanotubes
NGCs	Nerve guidance conduits
nHAp	Nano-hydroxyapatatite
OC	Osteocalcin
OP	Osteopontin
OSTRX	Osterix
P-15	PepGen P-15 peptide
PBMNCs	Peripheral blood mononuclear cells
PBS	Phosphate buffered saline
PCL	Poly-ε-caprolactone
PDGF	Platelet-derived growth factor
PDLCs	Periodontal ligament cells
PDO	Polydioxanone
PEG	Polyethylene glycol
PGA	Poly-glycolic acid
PGs	Proteoglycans
PHSRN	Pro-His-Ser-Arg-Asn amino acid sequence
PLA	Polylactic acid
PLGA	Poly(lactic-co-glycolic acid)
PLL	Poly-L-lysine
PLLA	Poly(L-lactic acid)
PNS	Peripheral nervous system
Que	Quercetin
RADARADARADARADA	Arg-Ala-Asp-Ala-Arg-Ala-Asp-Ala-Arg-Ala-Asp-Ala-Arg-Ala-Asp-Ala amino acid sequence
REDV	Arg-Glu-Asp-Val amino acid sequence
RGD	Arg-Gly-Asp peptide sequence
rhBMP-2	Recombinant human bone morphogenetic protein-2
rhBMP-7	Recombinant human bone morphogenetic protein-7
rhEpo-CNPs	Recombinant human erythropoietin-loaded chitosan nanoparticles
Rhodamin-FN	Rhodamin labeled fibronectin
RUNX2	Runt-related transcription factor-2
SAMs	Self-assembled monolayers
SAPs	Self-assembly peptides
SCs	Schwann cells
SDF-1α	Stromal cell-derived factor-1
SE	Soluble elastin
SEM	Scanning electron microscope
SIKVAV	Ser-Ile-Lys-Val-Ala-Val amino acid sequence
SMCs	Smooth muscle cells
TE	Tissue engineering
TGF-β	Transforming growth factor-β
UCB-MSCs	Umbilical cord blood-derived mesenchymal stem cells
VEGF	Vascular endothelial growth factor
VPGVP	Val-Pro-Gly-Val-Pro amino acid sequence
WVTR	Water vapor transmission rate
YIGSR	Tyr-Ile-Gly-Ser-Arg amino acid sequence
ZnNPs	Zinc nanoparticles
α-TCP	Alpha-tricalcium phosphate
β-TCP	Beta-tricalcium phosphate
